# Unilateral Maxillary Sinus Actinomycosis with a Closed Oroantral Fistula

**DOI:** 10.1155/2017/7568390

**Published:** 2017-03-02

**Authors:** Jason E. Cohn, Mark Lentner, Hui Li, Matthew Nagorsky

**Affiliations:** ^1^Department of Otolaryngology-Head and Neck Surgery, Philadelphia College of Osteopathic Medicine, Philadelphia, PA, USA; ^2^Department of Otolaryngology-Head and Neck Surgery, Drexel University College of Medicine, Philadelphia, PA, USA; ^3^Department of Pathology and Laboratory Medicine, Drexel University College of Medicine, Philadelphia, PA, USA

## Abstract

Actinomycosis is a bacterial infection due to* Actinomyces israelii*, a gram-positive, anaerobic organism that normally affects the cervicofacial region. However, facial injury or trauma (i.e., dental procedures) can allow this bacteria to inhabit other regions. There have been rare reports of actinomycosis of the paranasal sinuses. We present a case of a 50-year-old female who originally presented with a suspected oroantral fistula who subsequently was found to have actinomycosis involving her right maxillary sinus. Additionally, the dental extraction site revealed no connection with the maxillary sinus. We discuss the diagnostic approach and management of this patient as it relates to the limited existing literature.

## 1. Introduction

Actinomycosis is a bacterial infection due to* Actinomyces israelii*, a gram-positive anaerobic organism. Actinomyces normally exists in the aerodigestive flora and only crosses mucosal surfaces by means of facial injury or trauma [1]. Most commonly, this will occur as a result of a dental procedure, such as extraction or dental implants [2]. Actinomycosis usually occurs in 4 regions: cervicofacial, thoracic, abdominal, and genital [3]. Most cases present as a cervicofacial draining sinus near the mandible [4]. Rarely, an infection can involve the nose and paranasal sinuses. Most commonly you will see the maxillary sinus involved; however, you can also see ethmoid and sphenoid sinus involvement [2].

## 2. Case Report

**Figure 1:**
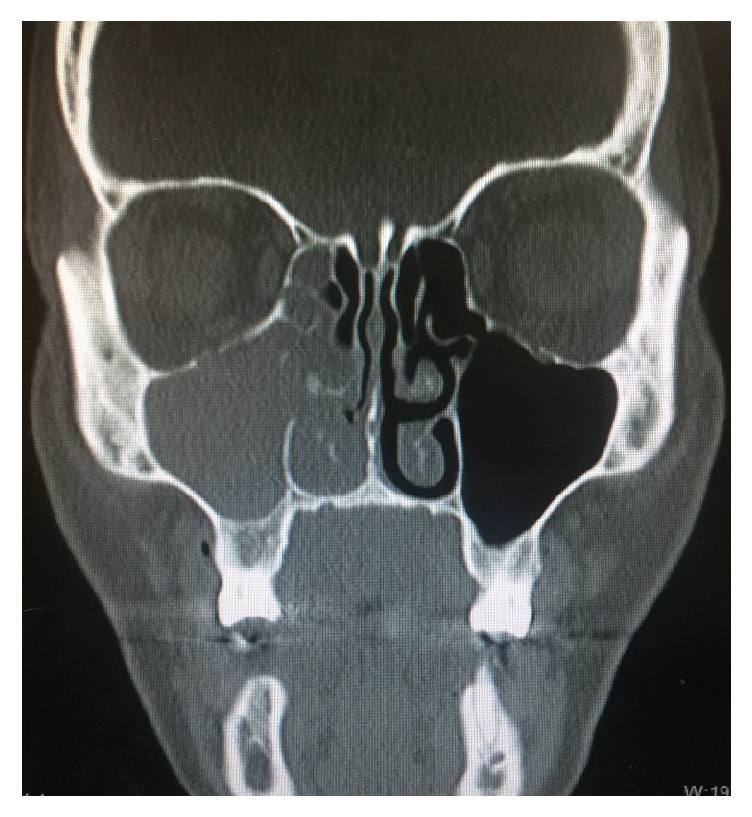
A coronal CT image demonstrating complete opacification of the right maxillary sinus with soft tissue extending through the ostiomeatal complex and into the right nasal cavity.

A 50-year-old female presented with right facial pain and tenderness since a dental extraction. Two weeks before she had her right upper molar extracted. Shortly after this procedure, she began draining foul-smelling and tasting green mucus. She also described mild right facial swelling and right maxilla numbness, as well as right ear fullness, pressure, and hearing loss. She denied fevers and chills. On examination, her right maxilla was tender to palpation and there was granulation tissue at the level of the right upper molar. However a distinct oroantral fistula was not appreciated. On nasal endoscopy, green mucus was visualized at the right ostiomeatal complex. At this time, she was advised to have a CT scan of the sinuses to further evaluate her sinus disease. CT scan of the sinuses demonstrated complete opacification of the right maxillary sinus with soft tissue extending through the ostiomeatal complex and into the right nasal cavity (Figure 1). There was also mucosal disease in the right ethmoid, frontal, and sphenoid regions. There was no left-sided sinonasal disease. Previously, the patient was treated with several courses of antibiotics with her primary care physician and, however, still experienced symptoms. At this point, she was brought to the operating room for right functional endoscopic sinus surgery (FESS) with possible closure of an oroantral fistula.

**Figure 2:**
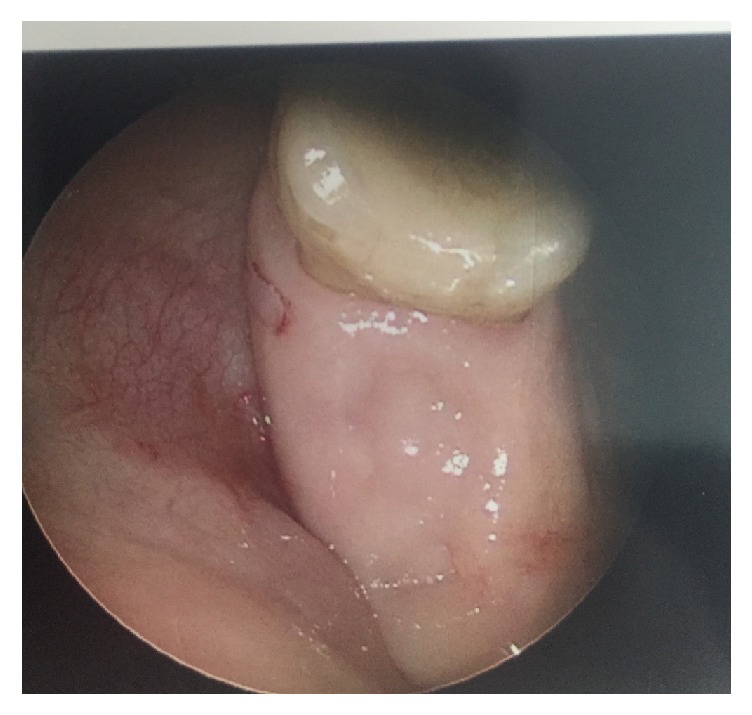
Intraoperative palpation of the dental extraction site revealing no opening into the maxillary sinus (oroantral fistula).

**Figure 3:**
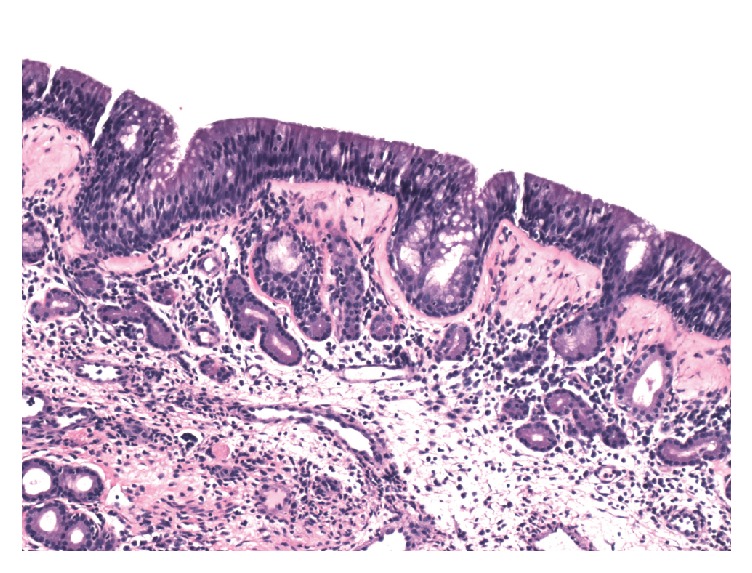
A histologic specimen of respiratory mucosa with thickened basement membrane and mild chronic inflammation.

Intraoperatively, she was found to have yellow-green mucopurulent material and edema in the right middle meatus extending to the nasopharynx. There was no visible disease on the left side. Palpation of the dental extraction site revealed no opening into the maxillary sinus (Figure 2). The patient underwent a right middle turbinectomy, maxillary antrostomy, and anterior and posterior ethmoidectomy. The specimens taken from the right maxillary sinus were irregular fragments of pink-tan fibrous soft tissue measuring 1.8 × 1.3 × 0.8 centimeters (cm) in aggregate. Yellow-green mucopus expressed from the right maxillary sinus was collected for aerobic and anaerobic culture. Shortly after the procedure, the microbiologist indicated that the intraoperative anaerobic culture revealed gram-positive, rod-shaped bacteria with fungus-like branching of hyphae. These rare findings were consistent with* Actinomyces*. However, the pathology specimens did not show any presence of* Actinomyces*. The only histologic finding was chronic inflammation and thickening of the basement membrane in the respiratory mucosa (Figure 3).

**Figure 4:**
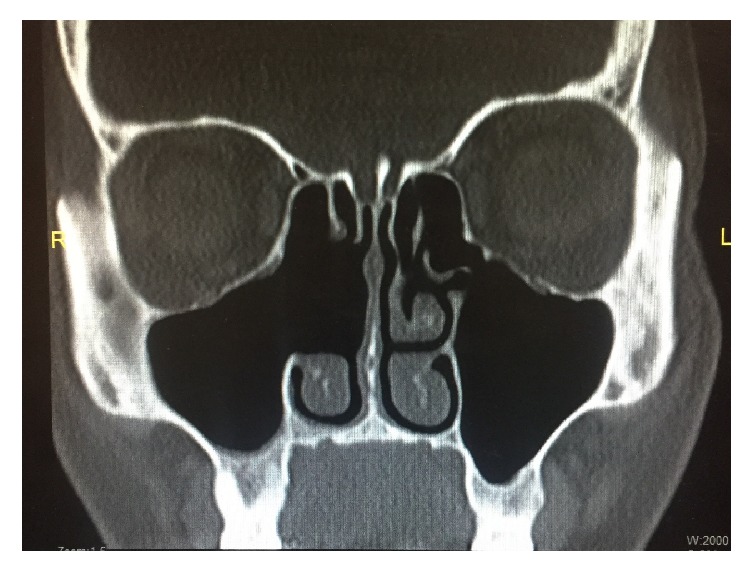
A postoperative coronal CT image demonstrating well-aerated right maxillary and ethmoid sinuses without mucosal thickening with a patent ostiomeatal complex after right maxillary antrostomy, middle turbinectomy, and anterior and posterior ethmoidectomy.

Treatment of actinomycosis involves surgical debridement as well as long-term antibiotic therapy, typically penicillin [5]. Therefore, this patient underwent FESS as well as treatment with long-term antibiotics. In the immediate postoperative period, the patient was seen weekly for nasal endoscopy with debridement on three separate occasions and was treated with penicillin-VK 500 milligrams (mg) four times per day. However, this therapy was discontinued early after a total of 7 weeks treatment due to gastrointestinal upset and dizziness. Three weeks after the penicillin was discontinued, the patient began to experience right maxillary sinus pressure again. At this time, the patient was reevaluated by our surgical team as well as an infectious disease specialist. Doxycycline 100 mg twice a day was instituted and has continued to date. Currently, she is doing well with no active sinonasal disease. She no longer reports sinus pressure or drainage. On nasal endoscopy, her right ostiomeatal complex is widely patent without mucopurulent drainage. A postoperative CT scan revealed a normal maxillary antrum without fluid collection or mucosal thickening (Figure 4).

## 3. Discussion

Actinomycosis of the paranasal sinuses was first described by Ponfick in 1882 and then specifically in the maxillary sinus by Stanton in 1966 [6]. Actinomycosis of the paranasal sinuses remains rare and has only been further demonstrated by a small number of case reports. Occasionally, patients can become susceptible to invasive sinonasal actinomycosis and present with headache, visual changes, and cranial nerve palsy [4, 7].

It has been shown that the presence of an oroantral fistula can predispose one to actinomycosis of the paranasal sinuses [5, 8]. Our patient was originally referred to our group for an oroantral fistula. However, examination in the office as well as the operating room revealed spontaneous closure of the fistula. Despite this finding, sinonasal cultures revealed growth of* Actinomyces*. Therefore, it is important to consider additional etiologies for actinomycosis in this patient. For example, it has been shown that sinus hypoxia can occur through blockage of sinus ostia causing an anaerobic environment for* Actinomyces* [1, 4]. This patient had significant edema surrounding her middle meatus; therefore this theory is plausible. Overall, this patient demonstrated both mechanisms of pathophysiology. The patient first experienced odontogenic sinusitis from the penetration of oral pathogens into the sinonasal cavities via an oroantral fistula. Once this fistula closed,* Actinomyces* was able to flourish in an anaerobic environment due to local tissue hypoxia from paranasal sinus inflammation.

The diagnosis of actinomycosis is accomplished with a thorough history and physical examination, the presence of yellow sulphur granules on specimens, and specific findings on radiographic imaging [5]. CT findings suggestive of actinomycosis include opacification, unilateral lesion, mucosal thickening, thickening of bone walls, focal areas of bone destruction (especially of the medial wall), and calcifications [9]. This case was unusual because of the pathogen isolated and the lack of several typical radiologic features. The CT clearly demonstrated right-sided opacification of all paranasal sinuses, soft tissue occupying the right nasal cavity, and right paranasal sinus mucosal thickening (particularly in the maxillary sinus). However, thickening of bone walls, focal areas of bone destruction, and calcifications were not seen. Based upon the clinical course of this patient, we recommend the usual 6-month treatment with penicillin-VK 500 mg four times per day. However, prescribing physicians should be aware that there are potential side effects and poor patient compliance due to frequent dosing. This patient is improving significantly with doxycycline; therefore, that is a viable option. Due to the improvements seen both clinically and radiologically, we did not feel the need to order further testing such as magnetic resonance imaging (MRI). However, MRI can be useful to clinicians to evaluate soft tissue invasion [7].
